# Cell Penetrating Peptide Derived from Human Eosinophil Cationic Protein Decreases Airway Allergic Inflammation

**DOI:** 10.1038/s41598-017-12390-8

**Published:** 2017-09-27

**Authors:** Lin-shien Fu, Yu-Rou Wu, Shun-lung Fang, Jaw-ji Tsai, Heng-kuei Lin, Yee-jun Chen, Ting-Yu Chen, Margaret Dah-Tsyr Chang

**Affiliations:** 1 Pediatric Department, Taichung Veterans General Hospital, Taichung, Taiwan; 2 Medical Research Department, Taichung Veterans General Hospital, Taichung, Taiwan; 3 Institute of Molecular and Cellular Biology, National Tsing Hua University, Hsinchu, Taiwan; 4 Department of Life Science, National Tsing Hua University, Hsinchu, Taiwan; 5 Pediatrics Department, National Yang-Ming Medical University, Taipei, Taiwan

## Abstract

Cell penetrating peptide derived from human eosinophil cationic protein (CPPecp) is a 10-amino-acid peptide containing a core heparan sulfate (HS)-binding motif of human eosinophil cationic protein (ECP). It binds and penetrates bronchial epithelial cells without cytotoxic effects. Here we investigated airway-protective effects of CPPecp in BEAS-2B cell line and mite-induced airway allergic inflammation in BALB/c mice. In BEAS-2B cell, CPPecp decreases ECP-induced eotaxin mRNA expression. CPPecp also decreases eotaxin secretion and p-STAT6 activation induced by ECP, as well as by IL-4. *In vivo* studies showed CPPecp decreased mite-induced airway inflammation in terms of eosinophil and neutrophil count in broncho-alveolar lavage fluid, peri-bronchiolar and alveolar pathology scores, cytokine production in lung protein extract including interleukin (IL)-5, IL-13, IL-17A/F, eotaxin; and pause enhancement from methacholine stimulation. CPPecp treated groups also showed lower serum mite-specific IgE level. In this study, we have demonstrated the *in vitro* and *in vivo* anti-asthma effects of CPPecp.

## Introduction

Asthma is a chronic, ongoing lung disease marked by acute flare-ups or attacks of difficulty with breathing such as shortness of breath, cough, chest tightness, and wheezing sound, and characterized by reversible obstruction of airway hyper-responsiveness (AHR) and airway inflammation in which eosinophil, lymphocytes, and neutrophil infiltrate airway^1,2^.

B cells, T-helper 2 (Th2) cells and Th2 related mediators play key roles in inflammatory responses in allergic asthma. Among which interleukin (IL)−4 is a cytokine regulating development of allergic inflammation. It is associated with induction of Th2 differentiation as well as allergen specific IgE secretion by B cells^3^. In addition, IL-4 increases eotaxin expression through activation of Janus family tyrosine kinases and phosphorylation of signal transducer and activator of transcription (STAT), hence promotes eosinophilic inflammation by inducing eosinophil chemotaxis^4^.

In eosinophil, several granular proteins including major basic protein (MBP), eosinophil cationic protein (ECP), eosinophil peroxidase (EPX), and eosinophil-derived neurotoxin (EDN) have been suggested to contribute to not only AHR but also remodeling that is associated with severe asthma^2^. ECP secreted from activated eosinophil contributes to elimination of invading microbes such as parasites and viruses^5^. In addition, together with other proteins secreted from eosinophil, ECP causes damage to the airway, a common feature of airway allergic inflammation in asthma^6^, as extracellular deposits of ECP are found in tissues undergoing eosinophilic inflammation resulting in tissue damage^7^. ECP interacts with mammalian cells by binding to carbohydrates on the cell surface^8^ with high affinity for structures containing glycosaminoglycan (GAG), in particular, heparan sulfate proteoglycans (HSPGs), leading to internalization of ECP through HS-mediated and raft-dependent macropinocytosis along with intracellular trafficking from macropinosome to late endosome/lysosome^7^. Hence ECP triggers cytotoxicity through destabilization of lipid membranes of target cells and apoptosis^9^. In addition, *in vivo* models have demonstrated that ECP preferentially targets tracheo-epithelial cells in rat mainly due to abundant HSPG expression on cell surface^10^, and radiolabeled ECP is able to image HS expression to predict allergic lung inflammation in an asthma mouse model^11^. However, cytotoxicity of ECP is severely reduced in HS-deficient cell lines, *in vivo* targeting of ECP to lung tissues is significantly diminished in the presence of heparanase, implying critical roles of HSPGs in ECP function.

Interestingly, a sequential 10-amino-acid peptide NYRWRCKNQN residing in ECP^8^, also named as CPPecp, has been identified to accommodate a core HS-binding motif^10^. Besides its binding to cell surface HS, this segment possesses cell penetrating character by HS-mediated energy-dependent endocytosis, lipid-raft endocytosis and macro-pinocytosis^8^. CPPecp binds to human bronchial epithelial BEAS-2B cells yet exerts no cytotoxicity as ECP does^8^. Moreover, biodistribution of CPPecp *in vivo* mainly accumulates in bronchus and lung epithelial tissues employing intranasal inhalation and intravenous injection in BALB/c mice^8^.

GAGs are anchored on cell surface, as a signaling co-receptor, binding to multiple ligands, and promoting receptor-ligand interactions that mediate cell growth, motility, and immune response^12^. Among various GAGs HS has been identified in all animal tissues. As a consequence of binding to a receptor, HS can recruit GAG-binding natural receptor ligands, for example, IL-4^13^, IL-5^14^, IL-6^15,16^ and IL-13^17^ to promote downstream bioactivities^18^.

Based on available *in vitro* and *in vivo* data, we hypothesized that CPPecp with GAG moiety-binding characteristics may protect cells from airway allergic inflammation by interfering binding of soluble mediators to GAGs on cell surface. Hence, we initiated *in vitro* experiments to examine cellular functions of CPPecp on IL-4/ECP effects on human bronchial epithelial BEAS-2B cell line. In addition, animal study was established to investigate *in vivo* effects of CPPecp on mite-induced airway allergic inflammation in BALB/c mice.

## Results

### *In vitro* studies

#### CPPecp decreased eotaxin (CCL11) transcript stimulated by ECP

**Figure 1:**
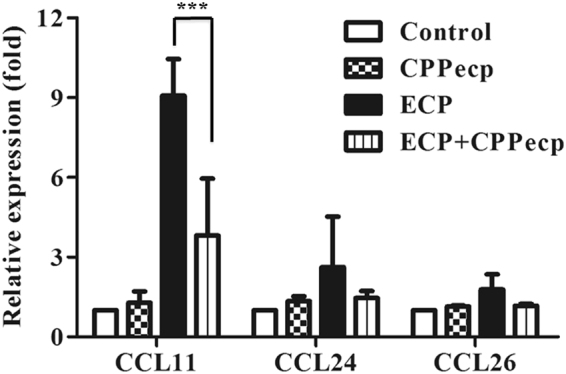
Quantitative analysis of indicated chemokine for eosinophil transcripts regulated by ECP and CPPecp CPPecp reduced mRNA expression of ECP-induced eotaxins. BEAS-2B cells were starved for 24 h, followed by stimulation with refolded 5 μM ECP or 5 μM CPPecp or co-treatment with 5 μM ECP and 5 μM CPPecp at 37 °C for 6 h. Quantitative RT-PCR analysis of eotaxin mRNA in presence of CPPecp in BEAS-2B cells. These data were expressed as relative fold with mock-treated set as one. The data represented at least three independent experiments and the error bar was shown as SD. ***p < 0.001.

BEAS-2B cells were separately cultured in serum-free medium in the presence of 5 μM ECP alone, co-treatment of 5 μM CPPecp for 6 h to monitor eotaxin gene modulation using real-time quantitative PCR. Untreated BEAS-2B cells incubated at the same condition for 6 h were used as negative controls. mRNA quantities of CCL11, CCL24, and CCL26 in treated groups of BEAS-2B cell were shown in Fig. 1. The transcript of CCL11 was stimulated 9-fold by 5 μM ECP, which was decreased 57% upon co-treatment with 5 μM CPPecp for 6 hours (*p* < 0.0001). However, no significant change in CCL24 or CCL26 transcript was observed.

#### CPPecp decreased eotaxin (CCL11) secretion stimulated by ECP or IL-4

**Figure 2:**
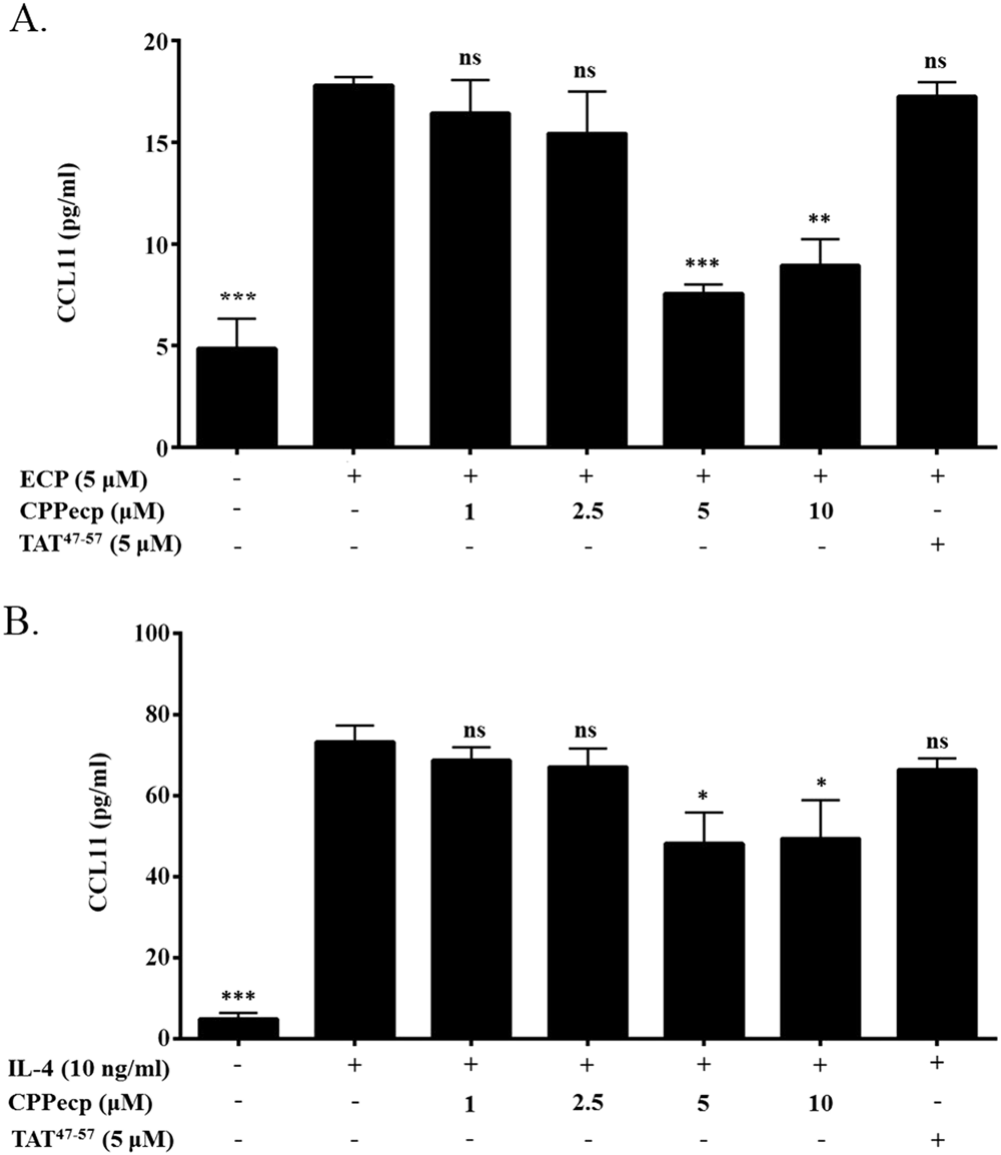
(**A**) CCL11 secretion in CPPecp and ECP co-treated BEAS-2B cells. BEAS-2B cells were starved with serum free medium for 24 h, followed by stimulation with ECP. Cells were stimulated with PBS as negative control and ECP in the absence of CPPecp or presence of 1, 2.5, 5, and 10 μM CPPecp at 37 °C for 24 h. Cells were stimulated with ECP in the presence of 5 μM TAT. CCL11 protein level was determined by ELISA kit employing CCL11 antibody. The data represented mean ± SD of at least three independent experiments. ***p < 0.001; **p < 0.01. (**B**) CCL11 expression in CPP*ecp* and IL-4 co-treated BEAS-2B cells. BEAS-2B cells were starved with serum free medium for 24 h, followed by stimulation with PBS or 10 ng/ml IL-4. Cells were stimulated with 10 ng/ml IL-4 in the absence or presence of 1, 2.5, 5 and 10 μM CPPecp at 37 °C for 24 h. Cells were stimulated with IL-4 in the presence of 5 μM TAT. CCL11 protein level was determined by ELISA kit employing CCL11 antibody. The data represented mean ± SD of at least three independent experiments. ***p < 0.001. *p < 0.05.

Eotaxin secretion from BEAS-2B cells was significantly enhanced 3.5-fold by 5 μM ECP (*p* < 0.0001), which was suppressed 58% and 50% by co-treatment with respectively 5 μM (*p* < 0.001) and 10 μM CPPecp (*p* < 0.01), but not 5 μM peptide derived from the transactivator of transcription of human immunodeficiency virus (TAT^47–57^ peptide), as shown in Fig. 2A. Likewise, IL-4 treatment stimulated even more secretion 9.5-fold of eotaxin from BEAS-2B cell (*p* < 0.001), which could be suppressed 34% and 33% by co-treatment with respectively 5 and 10 μM CPPecp (*p* < 0.05), as shown in Fig. 2B.

#### CPPecp decreased STAT6 phosphorylation induced by ECP or IL-4

**Figure 3:**
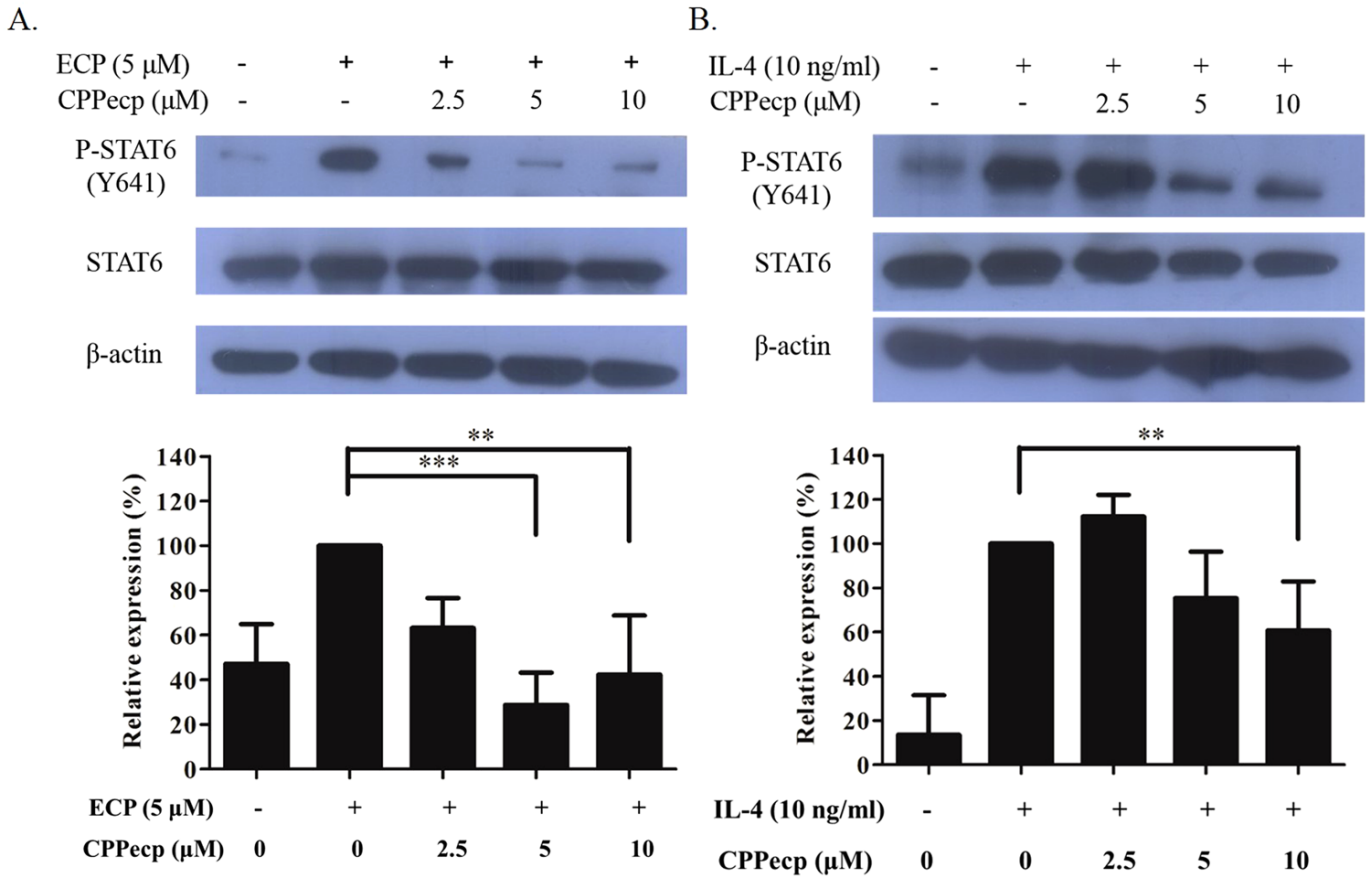
(**A**) CPPecp decreases ECP stimulated STAT-6 phosphorylation in BEAS-2B cells. BEAS-2B cells were treated with 5 μM ECP alone and in the absence or presence of CPPecp at 2.5, 5 and 10 μM for 15 min. Upper panel: Changes in protein levels of p-STAT6/STAT6 were determined by Western blotting. Lower panel: Intensity of protein band from control and treated cells was normalized to β-actin signal and the difference in protein expression at each concentration was expressed as percentage of p-STAT6 level stimulated by 5 μM ECP. Values are expressed as mean ± SD for three experiments. ***p < 0.001, **p < 0.01. (**B**) CPPecp decreases IL-4 stimulated STAT-6 phosphorylation in BEAS-2B cell. BEAS-2B cells were treated with 10 ng/ml IL-4 alone and combined both 10 ng/ml IL-4 and 2.5, 5 and 10 μM CPPecp for 5 min. Upper panel: Changes in protein levels of p-STAT6/STAT6 were determined by Western blotting. Lower panel: Intensity of protein band from control and treated cells was normalized to β-actin signal and the difference in protein expression at each concentration was expressed as percentage of p-STAT6 level stimulated by 10 ng/ml IL-4. Values are expressed as mean ± SD for three experiments. **p < 0.01.

ECP and IL-4 were used to induce phosphorylation of STAT6 (p-STAT6) in BEAS-2B cells, separately. p-STAT6 was induced by refolded ECP within 15 min, and it was evidently diminished in the presence of CPPecp (Fig. 3A, upper panel). Quantitative analysis showed that p-STAT6/STAT6 ratio was respectively decreased 37%, 71%, and 58% by 2.5, 5 and 10 μM CPPecp upon co-treatment with ECP (Fig. 3A, lower panel).

Figure 3B showed that IL-4 induced p-STAT6 within 5 min, which was suppressed by co-treatment with 5 and 10 μM CPPecp (Fig. 3B, upper panel). Quantitative analysis also revealed that p-STAT6/STAT6 ratio was respectively decreased 25% and 39% by 5 and 10 μM CPPecp (Fig. 3B, lower panel), suggesting that CPPecp might interfere with STAT6 phosphorylation induced by both ECP and IL-4 in BEAS-2B cells.

### *In vivo* studies

#### In Der p sensitized BALB/c mice, those received intranasal CPPecp treatment before intra-tracheal injection of Der p had lower eosinophil count in broncho-alveolar lavage fluid (BALF)

**Figure 4:**
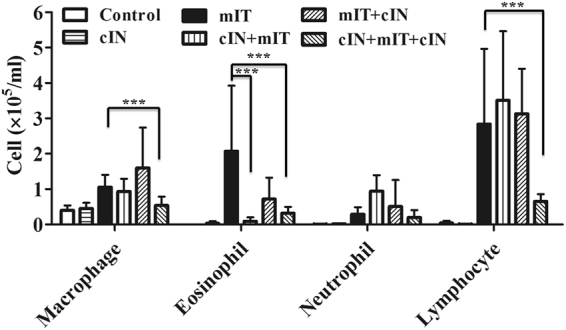
Cell counts of broncho-alveolar lavage fluid. The group cIN + mIT + cIN had lower macrophage, eosinophil and lymphocyte counts than mIT group (*p* < 0.001). The cIN + mIT had lower eosinophil count than mIT group (*p* < 0.001). ***p < 0.001.

Comparing with mite treated group (mIT), mice received intranasal CPPecp (cIN) from day 1 to 22 (cIN + mIT + cIN group) had respectively 51%, 85%, and 77% lower macrophage, eosinophil and lymphocyte counts than mIT group (*p* < 0.001), as shown in Fig. 4. The mice received cIN from day 1 to 15 (cIN + mIT group) had 95% lower eosinophil count than mIT group (*p* < 0.001), but 220% increase in neutrophil (*p* < 0.001) and 36% increase in lymphocyte (*p* = 0.06) in the BALF. However, the mice received cIN after intra-tracheal Der p administration on day 15 (mIT + cIN group) showed no difference in BALF cell count from the mIT group.

#### Intranasal CPPecp from day 1 to 22 (cIN + mIT + cIN group) had decreased histopathology scores in lung tissue compared to the mite treated BALB/c mice (mIT group)

**Figure 5:**
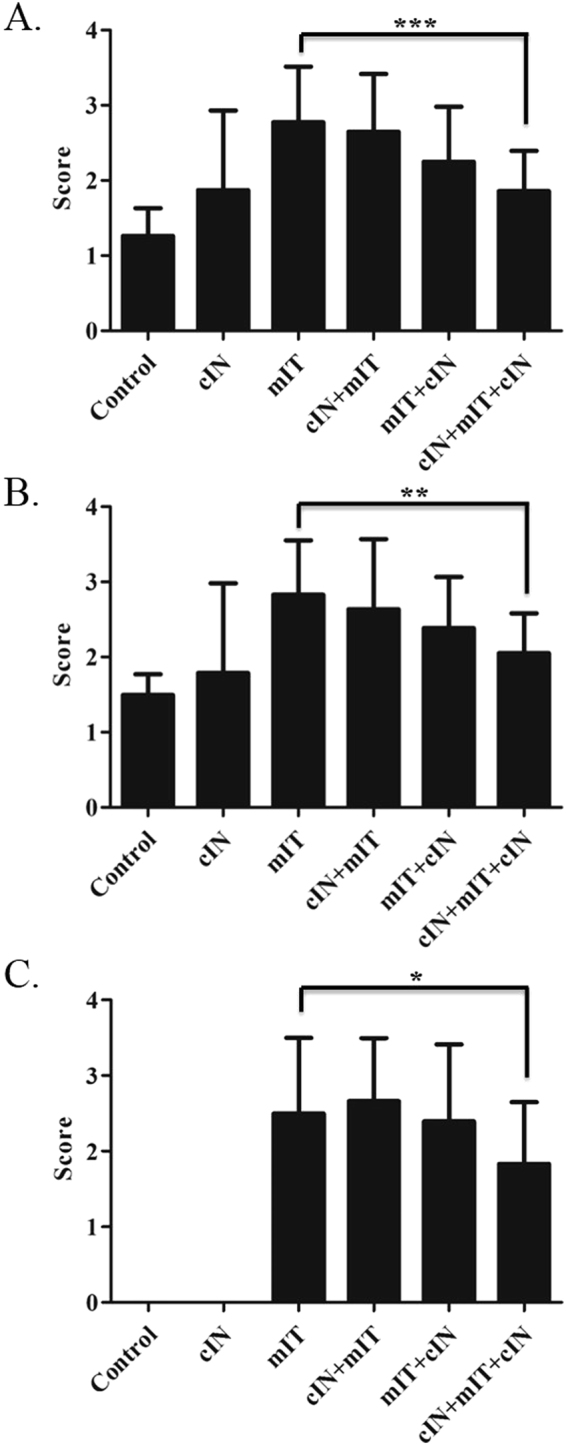
CPPecp suppresses airway inflammation pathology. (**A**) Scores for peri-bronchiolar and (**B**) alveolar inflammation were determined based on the histological sections by H&E staining. (**C**) Scores of hyperplasia goblet cell differentiation in medium-sized airways of mice were performed with periodic acid-Schiff (PAS) staining. *p < 0.05, **p < 0.01, ***p < 0.001.

Here we used the standard scoring system to interpret pathology score^19^ and goblet cell score^20^. To determine whether suppression of airway cellular inflammation was representative of a generally more-improved pathology, H&E staining of lung histological sections were compared among the six groups of mice, as demonstrated in Fig. 5. Comparing with mIT group, there was 33% lower peri-bronchiolar score in (*p* < 0.0001); and 28% lower alveolar pathology score (*p* < 0.009) in cIN + mIT + cIN group (Fig. 5A and B). The goblet cell scores of control and cIN groups were zero. The cIN + mIT + cIN group was had 27% lower score than mIT group (1.83 ± 0.92 *v.s*. 2.51 ± 0.85, *p* = 0.026), as shown in Fig. 5C. Pathology films are shown in supplementary information 2. Further H&E staining of specimen from heart, intestine, liver, spleen and kidney in all mice in these 6 groups detected no histopathology change was detected in these six groups.

#### Intranasal CPPecp treatment decreased IL-5, IL-13, IL-17A/F and eotaxin amount in lung protein extract of mite-induced asthma mice

**Figure 6:**
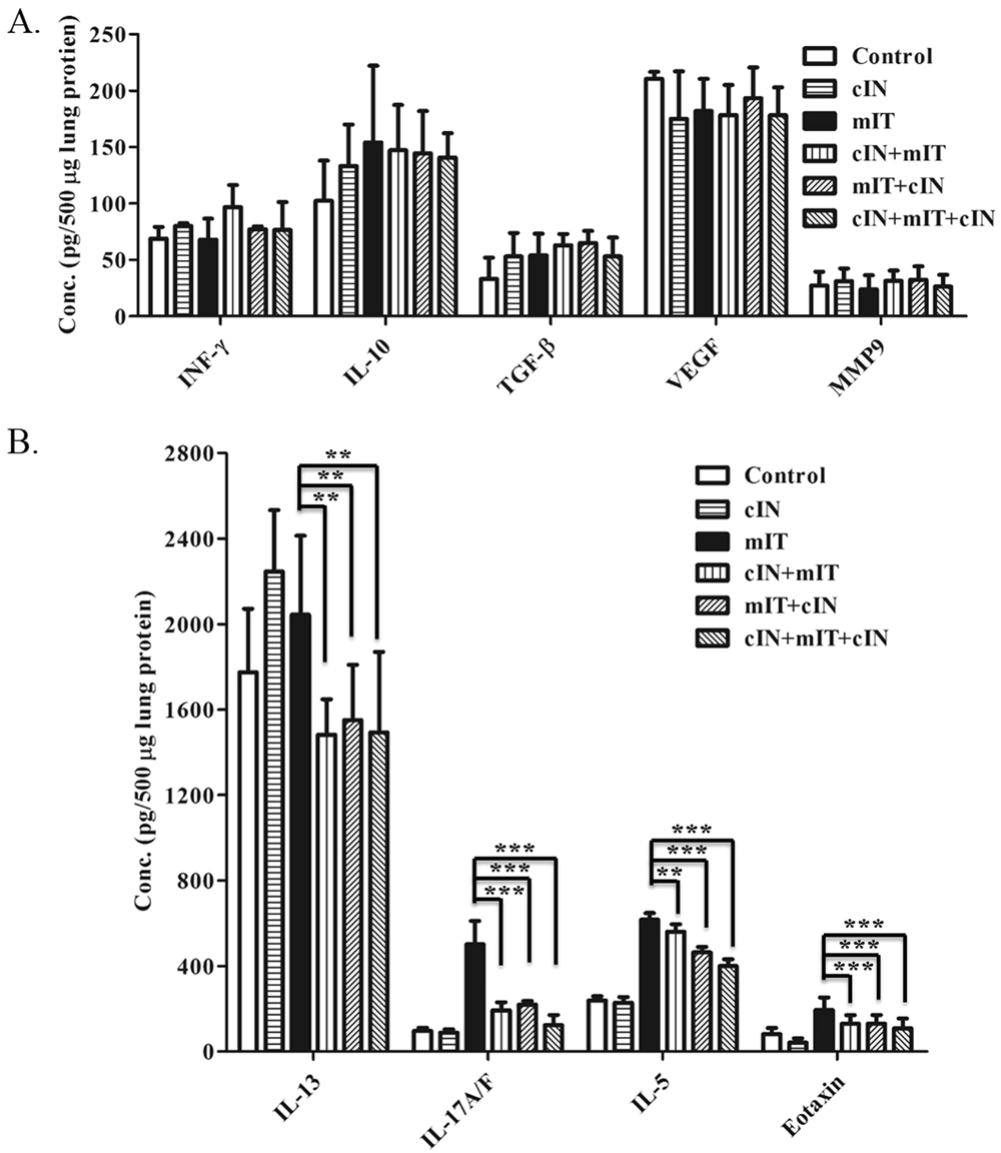
(**A**) Levels of IFN-γ, IL-10, TGF-β, VEGF, MMP9 in lung protein extracts. There was no difference among these cytokines six treatment groups. (**B**). Levels of IL-13, IL-17A/F, IL-5, and eotaxin levels in lung protein extracts. All values were compared with the mIT groupcIN + mIT, mIT + cIN, and cIN + mIT + cIN groups all had lower concentrations of IL-13 (all *p* = 0.004), IL-17A/F (all *p* < 0.000001), and eotaxin (*p* = 0.0007, 0.0008, and 0.0003, respectively). IL-5 levels were ower in mIT + cIN and cIN + mIT + cIN groups (*p* < 0.000001) as well as cIN + mIT group (*p* = 0.01). **p < 0.01, ***p < 0.0001.

We next analyzed the levels of several cytokines in lung protein extracts from mice in the six treatment groups. No difference in IFN-γ, IL-10, TGF-β, VEGF, or MMP9 concentrations was observed among the six groups as shown in Fig. 6A. Figure 6B illustrated that the treated groups including cIN + mIT, mIT + cIN, and cIN + mIT + cIN all showed about 24% lower concentrations of IL-13 than did the mIT group (all *p* = 0.004). The IL-17A/F levels in these groups were also 56% lower than that of the mIT group (all *p* < 0.000001). The IL-5 levels were respectively 24% and 35% lower in mIT + cIN and cIN + mIT + cIN groups (*p* < 0.000001), as well as 10% lower in cIN + mIT group (*p* = 0.001). These three groups, cIN + mIT, mIT + cIN, and cIN + mIT + cIN, also had about 34 to 44% lower concentrations of eotaxin than did the mIT group (*p* = 0.0007, 0.0008, and 0.0003, respectively).

#### Intranasal CPPecp treatment lowered pause enhancement (Penh) stimulated by methacholine

**Figure 7:**
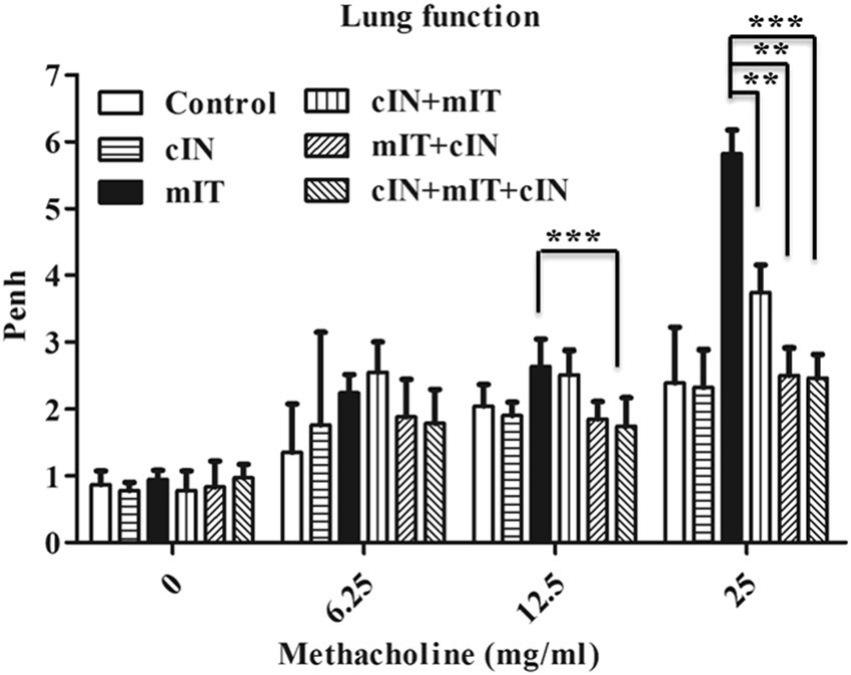
CPPecp effect on MCh triggered Penh in mice exposed to mite proteins. Penh was measured in six treatment groups after stimulation with 6.25, 12.5, or 25 mg/ml methacholine (MCh) for 1 min. The values are presented as a ratio with respect to the baseline Penh (before MCh stimulation) for each group. The mIT group showed a much higher Penh/baseline ratio change after stimulation with 12.5 and 25 mg/ml MCh than control, cIN + mIT + cIN and cIN groups (*p* < 0.001). The cIN + mIT and mIT + cIN groups showed lower stimulation than mite group treated with 25 mg/ml MCh (*p* = 0.002 and *p* = 0.003, respectively), but not with 12.5 mg/ml MCh. Thus, CPPecp clearly decreased allergen-induced AHR. *p < 0.05, **p < 0.01, ***p < 0.0001.

We followed established Penh evaluation methodology for BALB/c^21^. The changes in Penh in these six treatment groups after stimulation with increasing concentrations of methacholine (MCh) were shown in Fig. 7. The mIT group showed a much higher Penh/baseline ratio change after stimulation with 12.5 and 25 mg/ml MCh than control, cIN + mIT + cIN and cIN groups (*p* < 0.001). The cIN + mIT + cIN group had 59% lower Penh increase than the mIT group (*p* < 0.001). The cIN + mIT and mIT + cIN groups respectively showed 36% and 56% lower stimulation than mIT group treated with 25 mg/ml MCh (*p* = 0.002 and *p* = 0.003, respectively), but not with 12.5 mg/ml MCh.

#### Intranasal CPPecp treatment lowered Der p-specific serum IgE levels

**Figure 8:**
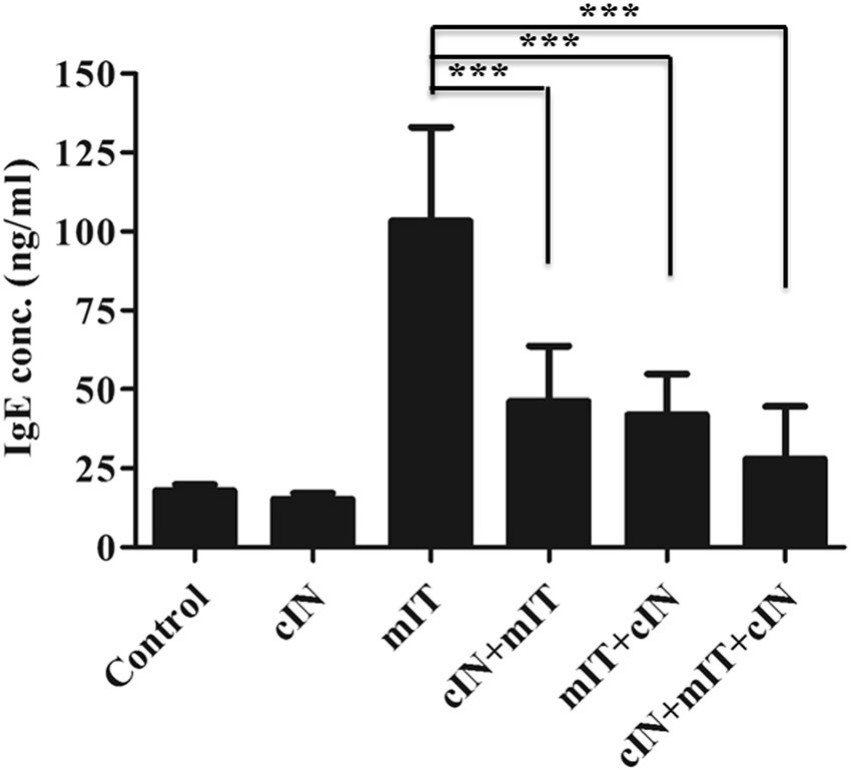
Serum Der p-specific IgE level on day 23 from mice in six groups. The mIT group had a higher Der p- specific IgE level than did the cIN + mIT, mIT + cIN, and cIN + mIT + cIN groups on day 23. *p < 0.05, **p < 0.01, ***p < 0.0001.

To further analyze inhibitory effect of CPPecp on mite sensitization, we measured serum Der p-specific IgE levels in mice from the six treatment groups. The sera were obtained on day 23 from IVC (Fig. 8). The mIT group had a higher IgE level (103 ng/ml) than did the cIN + mIT, mIT + cIN, and cIN + mIT + cIN groups on day 23 (all *p* < 0.01), approximately 2.5 to 3.6 fold higher than the CPPecp treated groups.

## Discussion

This pilot study suggests a protective effect of CPPecp on airway allergic inflammation in Der p-treated BALB/c mice. CPPecp decreased serum Der p-specific IgE level, BALF cell counts, pathology score, and cytokines including IL-5, IL-17A/F, IL-13, and eotaxin in lung protein extract. The Penh value stimulated by MCh as high as 25 mg/dL also decreased in the presence of CPPecp, suggesting that CPPecp reduced allergen-induced AHR. The group received intranasal CPPecp treatment from days 1 to 22 showed lower scores than pre-treatment and post-treatment groups. The microscopic examination of heart, intestine, liver, spleen and kidney from specimen from the six groups in this study showed no histopathological damage, indicating that CPPecp did not influence characteristic tissue properties.

ECP and CPPecp both bind to HSPGs on cell surface and have cell penetrating characters, as we have demonstrated previously^7–9^. CPPecp, the unique sequence derived from human ECP, has been demonstrated to be different from conventional GAG binding CPPs^8,22,23^. Recent reports showed that HS binding activity may interfere with *in vivo* functions of most HS-binding soluble modulators such as IL-4, IL-8, CCL11, and vascular endothelial growth factor (VEGF) in immune response^3,24^. IL-4 is a known GAG-binding cytokine^25^, and it binds components of the extracellular matrix^26,27^. den Dekker E. *et al*. have demonstrated that sulfated monocyte cell surface GAGs support IL-4 activity^13^, hence CPPecp may interfere with IL-4 binding to GAGs either on cell surface or in extracellular space. As demonstrated in our previous publication, CPPecp does not exert cytotoxic effect on BEAS-2B cell^8^. The present *in vitro* study revealed that CPPecp decreased ECP stimulated eotaxin transcript and secretion, and similarly, CPPecp decreased eotaxin secretion stimulated by IL-4. Both IL-4 and ECP are known to trigger activation of STAT6 with elevated level of p-STAT6 in target cells, and such phosphorylated expression in BEAS-2B cell was evidently decreased by co-treatment with CPPecp. Since p-STAT6 binds to promoter and subsequently activates eotaxin gene expression in human airway epithelia^28^ and lung fibroblast^29^, attenuation of p-STAT6 by CPPecp can be one explanation for decreased eotaxin production in Der p stimulated mice in the present study. In addition, Shamari *et al*. have also demonstrated that eotaxin elicit secretion of eosinophil associated ribonuclease (EAR) including human ECP and mouse ortholog EARs from eosinophil and associated cell free granules^30^. In airway inflammation model a vicious cycle of eotaxin and EARs secretion may possibly be reduced by the presence of CPPecp, leading to alleviation of cell damage.

Besides eotaxin, decreased p-STAT6 expression by CPPecp in airway epithelia also provides rational explanation for decreased IL-13 and IL-5 secretion in lung protein extract. Decreased local IL-13 production leads to lower serum Der p specific IgE level, implying that CPPecp involves Th2 immunomodulation. Effective therapies other than corticosteroids are important target for asthma management in considering side effects of steroid and versatile pathogenesis in asthma. Immunomodulation to decrease Th2 deviation is an important part in controlling allergic inflammation. Besides allergen peptides for immunotherapy, several peptides effective in treating asthma in animal model by modulating Th2 activity have been reported in recent years, for example, lipoprotein A-I peptide^31^, transglutaminase 2^32^ and STAT6 inhibition peptide (STAT6-IP)^33^. Wang *et al*. reported STAT6-IP, possessing Th2 modulation effect, decreased allergic inflammation in ragweed-induced asthma mice *via* intranasal administration^33^. STAT6-IP is composed of a HIV-TAT sequence as cell penetrating vehicle coupled to eight amino acids surrounding the phosphotyrosine residue at position 641 of STAT6. Now we have found that CPPecp, another effective peptide in reduction of STAT6 activation, given intranasally apparently decreases airway allergic inflammation in terms of reducing Der p specific IgE level as well as IL-13, IL-5, eotaxin secretion.

In recent years more and more evidences have revealed that allergic asthma to be a maladaptive Th17 response to airborne allergens; and such deviations start from dendritic cell in airway^34^. Th17, a distinct subset of T helper cell, secretes IL-17A/F. Th17 is differentiated from Th0 through the effect from cytokine IL-1β, IL-6, IL-21 and IL-23. The present *in vivo* study revealed CPPecp decreased IL-17A/F in lung protein extract. This effect can be explained by the fact that CPPecp can decrease inflammasome activation in human peripheral blood monocyte and dendritic cell line stimulated by Der p2 allergen^35^. Allergen can trigger pro-IL-1β production through a toll-like receptor (TLR)−4 mediated signal^36^. IL-1β creates a pro-inflammatory milieu with the production of IL-6 and chemokine which mobilize neutrophils and enhance Th17 cell differentiation in the lung^37^. These evidences further explain decreased IL-17A/F secretion by CPPecp treatment in Der p-induced airway inflammation.

Heparan sulfate (HS), variably-sulfated carbohydrate polymers, covalently attach to the core protein of HSPGs, are abundant on cell surface to form part of extracellular matrix (ECM)^38^. More and more evidences suggest that HS side chains of HSPGs modulate cytokine, chemokine, and cell surface adhesion molecule functions to coordinate the inflammatory processes^39–41^. Studies regarding asthma show that bronchial fibroblasts derived from asthmatic subjects have significantly higher levels of proteoglycans including small HSPGs^42^. In addition, HS side chains of HSPGs play a critical role in AHR and inflammation. Deficiency of endothelial HS attenuates allergic airway inflammation. Either lack of a gene encoding a key enzyme (*N*-deacetylase/*N*-sulfotransferase-1) involving in the biosynthesis of HS side chains, or anti-HS antibody treatment in lung endothelial cells significantly reduces AHR and the recruitment of inflammatory cells such as eosinophils, macrophages, neutrophils, and lymphocytes^43^. More recently,I^125^ labeled recombinant human ECP (rECP) has been demonstrated to bind to BALB/c mice; and the binding was much enhanced in ovalbumin sensitized asthma model^11^. Interestingly, the binding of ECP can be decreased by heparinase III pre-treatment in the BALB/c asthma model, strongly indicating that the *in vivo* binding target of ECP would be HS. In our previous *in vitro* study, CPPecp also binds HS as ECP does, cellular binding and subsequent internalization can be significantly decreased by the presence of soluble GAGs such as low molecular weight heparin (LMWH) and chondroitin sulfate C^7^. Our group has demonstrated the protective effect of intranasal LMWH treatment on Der p-induced airway allergic inflammation in terms of AHR, histopathology change, Der p-specific IgE level, and immune-modulation^44,45^. Heparin has been proved to be effective in preventing exercise-induced asthma^46^. So, those heparin mimics, and molecules interfering HS–protein interactions are potential anti-inflammatory compounds by competitively inhibiting the functions of HS side chains^47^.

In our previous studies we have clearly demonstrated the HSPG binding and internalization of CPPecp in BEAS-2B cell^7,8,10^; and the *in vivo* data showed venous injected CPPecp deposited in respiratory epithelia and intestinal villi^8^. It should be noted that our CPPecp possessing unique features in HS binding and membrane interaction among peptides of comparable sequences have been clearly demonstrated by nuclear magnetic resonance study^18^, together with *in vitro* and *in vivo* data in hand strongly supporting novel cytokine modulation activity of this multi-functional peptide.

In conclusion this study has demonstrated that CPPecp has protective effect of allergic airway inflammation *via* decreasing serum Der p-specific IgE, local production of IL-13/IL-5/eotaxin/IL17A/F and histopathology changes. Our current working mechanisms include decreased p-STAT6 expression in airway epithelium and inflammasome formation in antigen presenting cell. At molecular level the effect of CPPecp can be explained by the interaction with GAG, especially HS, leading to interference of HS-binding mediators. Taken together, CPPecp is a potential molecule for further development of novel strategy in managing airway inflammation.

## Methods

### Peptide synthesis

CPPecp was synthesized under standard solid-phase peptide synthesis protocol by Kelowna International Scientific Inc. (New Taipei City, Taiwan). First, covalently link the first amino acid onto resin, and sequentially add amino acids to the resin according to the order of NYRWRCKNQN. Then cleave peptide from the resin as well as remove side-chain protecting groups. Peptide purity (>90%) was assessed by analytical high-performance liquid chromatography. Peptide sequences were confirmed by electrospray ionization mass spectrometry performed by Kelowna International Scientific Inc.

### Expression and purification of ECP

Recombinant ECP was expressed and purified in *Escherichia coli* BL21 (DE3) CodonPlus^®^ (Novagen, USA) cells as described previously^48^. Briefly, a synthetic gene for human ECP was cloned into the pET23a expression vector, and the protein was purified from inclusion bodies. Proteins collected from inclusion bodies were dialyzed against refolding buffer (20 mM Tris, 0.5 M arginine, 0.2 mM glutathione disulfide, 2 mM EDTA, 10% glycerol, pH 8.5), and purified refolded ECP was concentrated by Amicon Ultra-15 (Millipore, USA) and stored in PBS at −80 °C until use.

### *In vitro* experiment

We used virus-transformed human bronchial epithelial cell line, BEAS-2B, for the *in vitro* study. We treated the cell line with different conditions including control, ECP (5 μM) or IL-4 (10 ng/ml) alone, co-treatment of CPPecp 1, 2.5, 5 and 10 μM, as well as TAT^47–57^ (sequence: GRKKRRQRRRP) 5 μM. We collected cell protein after incubation, and then measured pSTAT6/STAT6 by Western blot assay. We quantitated cytokine/chemokine transcripts by real-time PCR after 6-hr incubation of cells. Supernatants for eotaxin measurement by ELISA (R&D systems, USA) were collected from cultured cell for 24 hours. The minimum detectable dose (MDD) of human eotaxin is typically less than 5 pg/ml. The detail of methods is described in supplementary information.

### Animal study protocol

**Figure 9:**
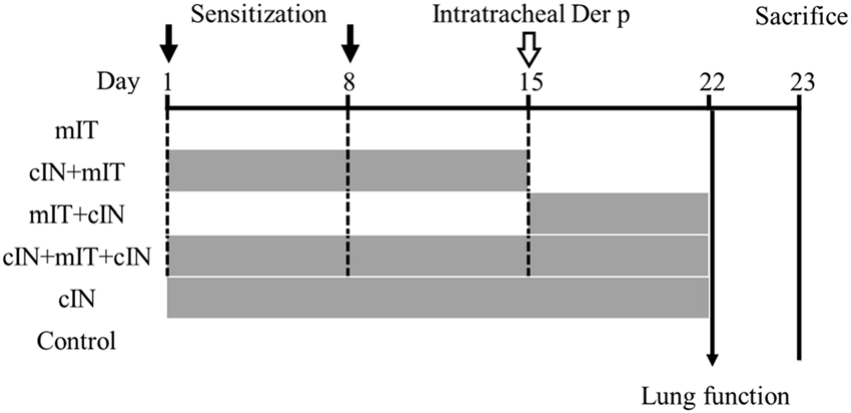
Animal Protocol: Brief summary of study protocol as 6 groups: 1. Control: intranasal PBS day 1–22, 2. cIN: intranasal CPPecp day 1–22, 3. mIT: day 1 and day 8 Der p subcutaneous sensitization + day 15 intra-tracheal Der p, 4. cIN + mIT: cIN day 1–15 + mIT, 5. mIT + cIN: mIT + day 15–22 cIN, 6. cIN + mIT + cIN: cIN day 1–22 + mIT.

As shown in Fig. 9, the male BALB/c mice were divided into six groups: 1) control, 2) mites delivered intratracheally (mIT) alone, 3) CPPecp intranasal 200uM, 10 μl (cIN) alone, 4) pre-treated group (cIN + mIT), 5) post-treated group (mIT + cIN), and 6) pre- and post-treated group (cIN + mIT + cIN). There were two separate experiments, the number of BALB/c mice was 5 in each group in one experiment. The protocol was briefly summarized as Fig. 9. All groups receiving mIT were immunized with 50 μl (40 μg) mite crude extract allergen on day 1 and day 8. Then we administered 10 μl of mite crude extract allergen (5 mg/ml, dissolved in phosphate-buffered saline [PBS], pH 7.4) intra-tracheally on day 15. The control group received PBS, pH 7.4 IN from day 1 to 22. The cIN and cIN + mIT + cIN groups received cIN from days 1 to 22, the cIN + mIT group received cIN from days 1 to 15 (before mIT), and the mIT + cIN group received cIN from days 15 (post-mIT) to 22. All mice were sacrificed on day 23. The detail about the preparation and procedure of mite immunization, mIT, cIN were described in supplementary information. The animal use protocol was reviewed and approved by Institutional Animal Care and Use Committee (IACUC Approval No: La-95279). The committee has checked this study by the following criteria: legal animal resource, the “Three Rs” principle, adequate raising and experiment place, and its full adherence to animal law in Taiwan.

### Studies on day 22 and day 23

We measured pause enhancement (Penh) one hour after the last intranasal administration of PBS or CPPecp.

On day 23, we sacrificed the mice by CO_2_ inhalation. Then, we did bronchoalveolar lavage of right side lung to analyze the cells in the fluid (BALF). We prepared tissue supernatant from left lung to quantify the amount of cytokine/chemokine including IL-5, IL-10, IL-13, IFN-γ, transforming growth factor-β (TGF-β), eotaxin, IL-17A/F, vascular endothelial growth factor (VEGF), and matrix metallopeptidase 9 (MMP9). We sampled blood from inferior vena cava (IVC) for Der p-specific IgE measurement. We sent samples from lung for pathology stain and scoring for inflammation, including H&E stain and PAS stain. We also sent specimen from heart, intestine, liver, spleen and kidney for checking if any pathological change in these visceral organs. The details of methods mentioned in this paragraph are described in supplementary information.

### Statistical analysis

Data were expressed as the mean ± standard deviation (SD). Analysis was performed with the ANOVA test for comparing multiple groups. Differences with a *p* value < 0.05 were considered significant. Graphs were performed using GraphPad Prism software (GraphPad Software, La Jolla, Calif). Analysis was carried out using Statistical Package for Social Sciences (version 10.1; SPSS, Chicago, IL, USA).

## Electronic supplementary material

Supplementary information
