# Supplementary material for: Cell Penetrating Peptide Derived from Human Eosinophil Cationic Protein Decreases Airway Allergic Inflammation
Source: Sci Rep. 2017 Sep 27;7:12352. doi: 10.1038/s41598-017-12390-8 (PMC5617860; doi:10.1038/s41598-017-12390-8)
Supplement: Supplementary file 1 — Supplementary information [file 41598_2017_12390_MOESM1_ESM.doc]

**Cell Penetrating Peptide Derived from Human Eosinophil Cationic Protein Decreases Airway Allergic Inflammation---Supplementary Information 1, 2 & 3**

Lin-shien Fu*‡, Yu-Rou Wu†, Shun-lung Fang†, Jaw-ji Tsai§, Heng-kuei Lin*, Yee-jun Chen*, Ting-Yu Chen† and Margaret Dah-Tsyr Chang†¶

From *Pediatric Department, §Medical Research Department, Taichung Veterans General Hospital, Taichung, Taiwan, †Institute of Molecular and Cellular Biology, ¶Department of Life Science, National Tsing Hua University, Hsinchu, Taiwan, ‡Pediatrics Department, National Yang-Ming Medical University, Taipei, Taiwan.

Address correspondence to: Lin-shien Fu, No.1650, Sec 4, Taiwan Blvd, Taichung, Taiwan 407. Fax: +886-4-23741359; Tel: +886-4-23592525 ext. 5910; E-mail address: [lsfu@vghtc.gov.tw](mailto:linshienfu@yahoo.com.tw), and Margaret Dah-Tsyr Chang, No. 101 Sec. 2, Kuang-Fu Road, Hsinchu, Taiwan 30013, Republic of China. Tel: +886-3-5742767; Fax: +886-3-5715934; E-mail address: [dtchang@life.nthu.edu.tw](mailto:dtchang@life.nthu.edu.tw)

**Supplementary information 1-Material and methods**

###### ***Animals***

Male BALB/c mice (6–8 weeks of age) were purchased from National Laboratory Animal Center (Nangang, Taipei, Taiwan) and were fed a commercial diet and water.

# Mite protein preparation

Crude extract of mite *Dermatophagoides pteronyssinus* (Der p) was purchased from Greer Lab (Lenior, NC, USA). The mite protein was isolated from the crude extract by homogenization with PBS using a glass homogenizer (Kontes Glass Company, Vineland, NJ, USA). The mite protein concentration was then determined using Bradford assay (Bio-Rad Protein Assay; Bio-Rad, Hercules, CA, USA).

# Immunization

Der p (1.6 mg/ml) was prepared and emulsified in complete Freund’s adjuvant (Sigma, St Louis, MO, USA) at a ratio of 1:1 [(v/v)] at 4 ºC. The mIT, cIN+mIT, mIT+cIN and cIN+mIT+cIN groups were immunized subcutaneously with 50 μl (40 μg) emulsified Der p on days 1 and 8.

# Intratracheal administration of mite protein

Der p (5 mg/ml) dissolved in PBS was given to each mouse on day 15. The mouse was placed in an upright position with its neck extended, and then 10 μl extract was directly administered to its trachea. Each mouse was anesthetized by inhaling isoflurane for approximately 1 min.

# Intranasal administration of CPPecp

CPPecp was dissolved in saline solution at a final concentration of 200 μM. 10 μl of CPPecp was then administered intranasally.

# Bronchoalveolar lavage fluid and measurement of cell count

On day 23 the mice were sacrificed in a CO_2_ chamber. The trachea was exposed and cannulated. The left main bronchus was tied with #4 suture thread. Two washings, each consisting of 1.0 ml PBS, pH 7.4, were introduced into the lungs *via* the cannula and withdrawn to collect the cells. Bronchoalveolar lavage fluid (BALF) from each wash was placed in polypropylene tubes on ice. The collected BALF was then centrifuged at 2,000 revolution/min for 7 min at 4 ºC. After supernatant removal, the cells were re-suspended in 1.0 ml of PBS. Total cell numbers were counted on a hemocytometer, and 1–5 x 10^3^ cells were spun onto glass microscope slides (cytospin 3; Shandon Scientific, Cheshire, UK). The cell slides were air dried for 24 h, fixed, and stained with Wright stain. Differential cell counts of at least 300 cells per slide were made according to morphological criteria. The number of cells recovered was calculated and expressed as absolute cell numbers.

# Histopathological analysis

The formalin-fixed left lung sections stained with hematoxylin and eosin (H&E) were assigned a unit value for alveolar, peri-bronchiolar, and total inflammation by computing the means of three independent scores for three randomly selected fields. Each section was independently and blindly interpreted by two physicians (Fu LS and Lin HK). We followed a previous scoring system for airway inflammation^1^. For evaluating the hyperplasia goblet cells, medium-sized airways were assessed in sections stained with periodic acid-Schiff (PAS) stain. Two reviewers (Fu LS and Chen YJ) independently and randomly scored 10 fields of each slide. Each lung was assigned a unit by computing the mean of the numerical scores. The numerical scores for the abundance of PAS-positive goblet cells^2^ in each airway were determined as follows: 0, 5% goblet cells; 1, 5–25%; 2, 25–50%; 3, 50–75%; 4, 75%. We also looked for pathological changes in the heart, intestine, kidney, spleen, and liver in these mice.

# Measurement of serum Der p–specific IgE

All BALB/c mice were sacrificed on day 23, and blood was immediately obtained from the inferior vena cava (IVC). All sera were stored in -80 ºC refrigerator and the level of serum Der p-specific IgE was measured by enzyme-linked immunosorbent assay (ELISA). Mite protein (5 μg) was coated on ELISA plates at 4 ºC overnight as previously described^3^. The anti-IgE antibody was purchased from B.D. (San Diego, CA, USA).

# Preparation of lung tissue supernatant

The left lung was homogenized in 1.0 ml cold PBS and preserved in iced bath before use. Lung homogenates were centrifuged at 20,000 x *g* for 5 min at 4 ºC. The lung tissue homogenate supernatants were then diluted in PBS to a final protein concentration of 500 μg/ml and stored at -80 ºC.

# Measurement of cytokine/chemokine in lung

Levels of cytokines in lung protein extract were evaluated using BD OptEIA^TM^ Set Mouse IL-5, IL-10, IL-13, IFN-γ, and transforming growth factor-β (TGF-β) kits (BD Bioscience, San Jose, CA, USA) and DuoSet mouse eotaxin, IL-17A/F, vascular endothelial growth factor (VEGF), and matrix metallopeptidase 9 (MMP9) kits (R&D, Minneapolis, MN, USA). The micro-plates were read at 450 nm with an ELISA reader (Thermo Lab systems, Waltham, MA, USA).

# Measurement of pause enhancement (Penh)

Airway responsiveness was measured in mice on day 22 (*i.e.*, 1 hour after the last intranasal inhalation) in conscious, spontaneously breathing mice using a whole-body plethysmography system (Buxco, Wilmington, NC, USA) as described before^4^. Briefly, the mice were individually placed in the chamber and allowed to settle for 3-5 min. The chamber-pressure-time wave was continuously measured *via* a transducer connected to a computer data acquisition system. After a baseline Penh reading for >3 min, the mice were serially exposed to increasing concentrations of nebulized methacholine (MCh) (0, 6.25, 12.5, and 25 mg/ml; Sigma, St Louis, MO, USA) in PBS for 1 min by inhalation. Penh values, which were measured as changes in enhanced pause, tidal volume, and breathing frequency (breaths/minute) for the first 3 min after the end of MCh nebulization, were averaged and used to compare responses among all six treatment groups.

# Cell and cell cultures

BEAS-2B cells (ATCC® number: CRL-9609™) were a human bronchial epithelial cell line infected with an adenovirus 12-SV40 virus hybrid (Ad12SV40). Cell lines were cultured in DMEM/F12 medium (Sigma-Aldrich) supplemented with 65 °C heat-inactivated 10% (v/v) fetal bovine serum (FBS) (Gibco/Invitrogen) and 1% (v/v) PSA (Penicillin, Streptomycine, and Amphotericin)(Biosera). The cells were incubated under a humidified atmosphere of 5% CO_2_ and 95% air.

For subculture, the cultured BEAS-2B cells in T75-flask or 100-mm dish were removed from humidified atmosphere to laminar flow. The aspirator was used to empty liquid medium covering cells and the cell monolayer was washed with 10 ml PBS without Ca^2+^/Mg^2+^. Then 1.5 ml trypsin/EDTA [0.05%/0.02% (w/v)] (BIOCHROM AG) was pipetted onto the washed cell monolayer and removed to 37 °C incubator for 5 min. After trypsin treatment, the cells were resuspended in 8.5 ml fresh serum-containing medium to inactivate trypsin, and 10-20 μl cells were seeded for cell count. The amount of 2x10^6^ cells was transferred to T75-flask or 1x10^6^ cells to 100-mm dish containing pre-warmed medium to a final volume of 10 ml. The BEAS-2B cells could be cultured and performed after two to three days.

# Western blotting analysis of STAT6 and STAT6 phosphorylation

Nearly confluent BEAS-2B cells were grown in FBS deprivation for 24 h. Cells were then stimulated in fresh FBS free medium with ECP (5 µM), IL-4 (10 ng/ml) or medium alone. At 5, and 15 min, the cells were washed twice with cold PBS, and total proteins were extracted with lysis buffer (1% NP-40, PMSF, 2 mM sodium orthovanadate, 0.1% sodium deoxycolate, and protease inhibitor cocktail (Roche, Penzberg, Germany). Harvested lysates were centrifuged to pellet cellular debris at 4 °C for 15 min. The supernatants were removed and stored at -80 °C. Protein lysate (30 µg) were loaded on 8% SDS-PAGE, followed by transferring to PVDF membranes (Merck Millipore, Germany). The blots were then blocked with 5% bovine serum albumin (BSA) in PBS containing 0.1% Tween 20 (PBST) at room temperature for 1 h, and then incubated at 4 °C overnight with antibodies specific for STAT6 and phosphorylated STAT6 (Y641) (Cell Signaling Technology, USA). After washing with PBST, the blots were incubated with goat anti-rabbit horseradish peroxidase (HRP) conjugated secondary antibodies and bands were visualized by enhanced chemiluminescence (ECL) reagents (Amersham Pharmacia,BaieD’Urfe, Quebec, Canada). β-actin was used as loading control.

## *Quantitation of chemokine (CCL11, 24, 26) transcripts by real-time PCR*

BEAS-2B cells were starved with serum free medium for 24 h, followed by stimulation with refolded 5 μM ECP or 5 μM CPPecp or co-treatment with 5 μM ECP and 5 μM CPPecp at 37 °C for 6 hours.

Total RNA is extracted with RNA TRIzol reagent (Invitrogen, Inc) and then incubated at 15 to 30 °C for 15 min. After that, 0.2 ml chloroform was added and shaken vigorously for 15 seconds and incubate at 15 to 30 °C for 2 to 3 min followed by centrifugation at 12,000 x *g* at 4 °C for 15 min. The aqueous phase was retained and 0.5 ml of isopropanol was added, incubated at 15 to 30 °C for 10 min and centrifuged at 12,000 x *g* at 4 °C for 10 min. After centrifugation, the RNA was precipitated, the supernatant was then removed and 1 ml 75% (v/v) ethanol was added to wash the RNA pellet. The samples were centrifuged at 7,500 x *g* at 4 °C for 5 min and the supernatant was removed. The RNA pellet was air dried at room temperature for 5-10 min, and then dissolved with 50 μl DEPC water. Purified RNA quality and concentration was assessed with a NanoDrop 1000 spectrophotometer.

One microgram of total RNA are then reversely transcribed into cDNA with Moloney murine leukemia virus (M-MLV) reverse transcriptase (Invitrogen) and oligo-dT primers (Invitrogen) in a 20 μl reaction. Gene glyceraldehyde-3-phosphate dehydrogenase (GAPDH) cDNA amplification serves as an internal control. Products of PCR are run on a 1.5% agarose gel and visualized by ethidium bromide staining. When needed, PCR products are purified from agarose gels with a QIAquick gel extraction kit (Qiagen Inc., Valencia, CA.). Direct sequencing of purified PCR products is performed using an ABI-PRISM model 377 sequencer (PE Applied Biosystems, Foster City, CA). Alternatively, PCR products are cloned with a pCR II-TOPO TA cloning kit (Invitrogen) and sequenced with T7 and Sp6 primers. The sequences are aligned with the corresponding database provided by the National Center for Biotechnology Information (Bethesda, MD). Quantitative real-time RT-PCR will be performed as follows. For one reaction, 2 microliter of cDNA, 10 microliter of 2X Power SYBR Green PCR Master Mix reagent (Applied Biosystems), 2 μl of primer pair and 6 μl of distilled water were mixed. PCR was performed by ABI StepOne Plus Real-Time PCR System. The C_t_ values of test genes were calculated by normalizing the C_t_ value of the internal house-keeping gene GAPDH. The gene expression level was calculated by the following formula for test mRNA expression:

△C_t_ test = C_t_ test-C_t_ GAPDH

expression level = 2^-(△C_t_ test-△C_t_ control)

Sequences of PCR primers for CCL11, 24, 26 were designed with Primer Express software (Applied Biosystems) for optimal product length, germinal center content, and T_m_ value.

## Human CCL11 Enzyme-link immune-sorbent assay (ELISA) kit

BEAS-2B cells were starved with serum free medium for 24 h, followed by stimulation with PBS or 5 μM ECP or IL-4 (10 mg/ml). Cells were stimulated with 5 μM ECP or IL-4 (10 ng/ml) in the absence or presence of 1, 2.5, 5 and 10 μM CPPecp at 37 °C for 24 h.

CCL11 ELISA kit (R&D) is an *in vitro* enzyme-linked immunosorbent assay for quantitative determination of human CCL11 concentration in cell culture supernatants. This assay employed an antibody specific for human CCL11 coated on a 96-well plate. The wells were incubated with 50 μl standard or sample for 2.5 hours at room temperature. After washing with 1x wash solution five times, 200 μl of CCL11 conjugate were added into each wells for 1 h. After washing, 100 μl TMB One-Step Substrate Reagent were added into each wells at room temperature for 30 minutes. 50 μl of Stop Solution were added into each wells and read at 450 nm by spectrophotometer immediately. Limit of detection in the assay was 5 pg/ml.

**References**

1 Ford, J. G. *et al.* Il-13 and IFN-gamma: interactions in lung inflammation. *J Immunol* **167**, 1769-1777 (2001).

2 Padrid, P. *et al.* Persistent airway hyperresponsiveness and histologic alterations after chronic antigen challenge in cats. *Am J Respir Crit Care Med* **151**, 184-193, doi:10.1164/ajrccm.151.1.7812551 (1995).

3 Lin, H. K. *et al.* Sodium sulfite aggravated allergic sensitization and airway inflammation in mite allergen sensitized BALB/c mice. *Hum Exp Toxicol* **30**, 1682-1689, doi:10.1177/0960327111398673 (2011).

4 Maeda, T. *et al.* Growth inhibition of mammalian cells by eosinophil cationic protein. *Eur J Biochem* **269**, 307-316 (2002).

**Supplementary information 2: Pathology**

H&E stain (light microscope 100x)


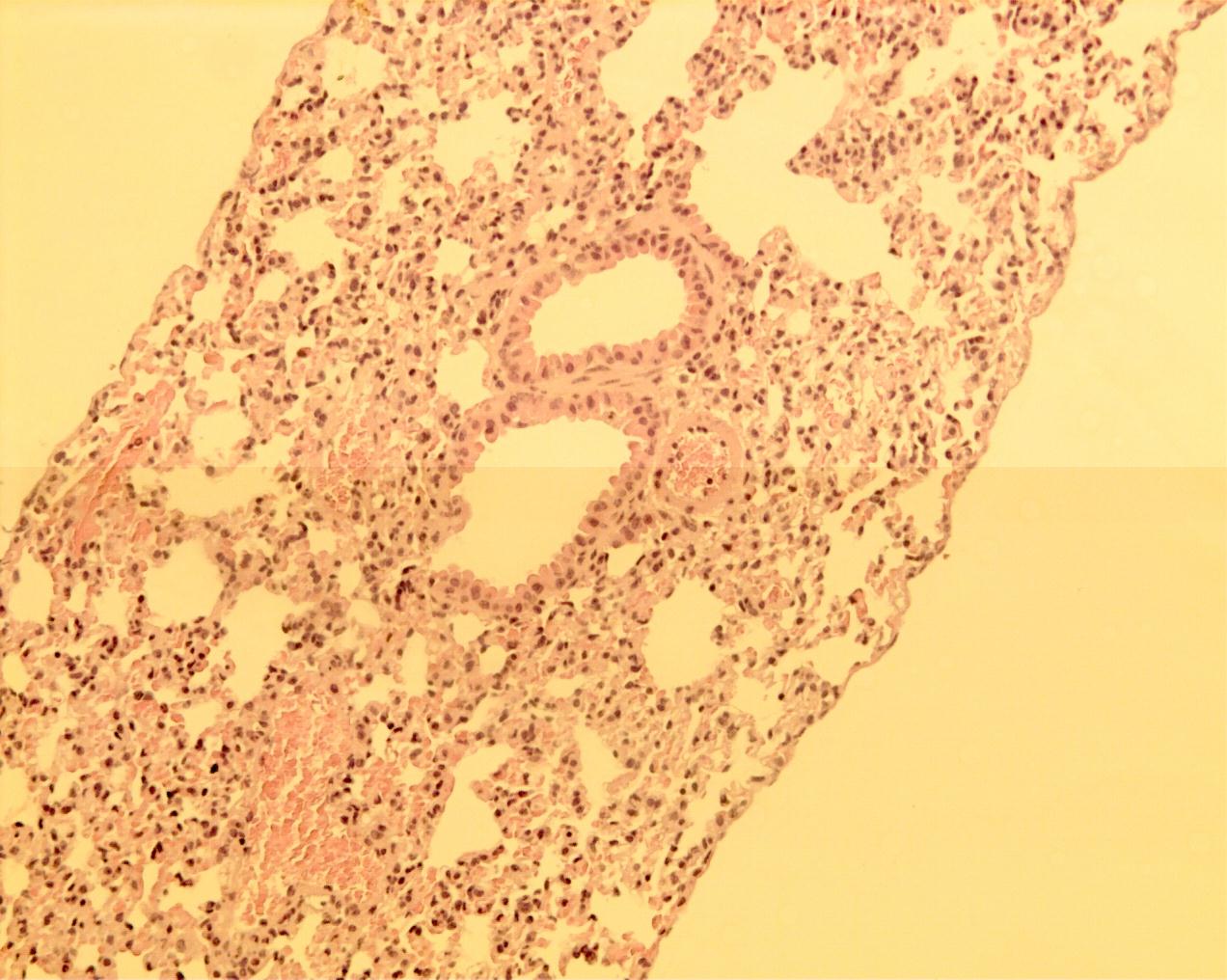

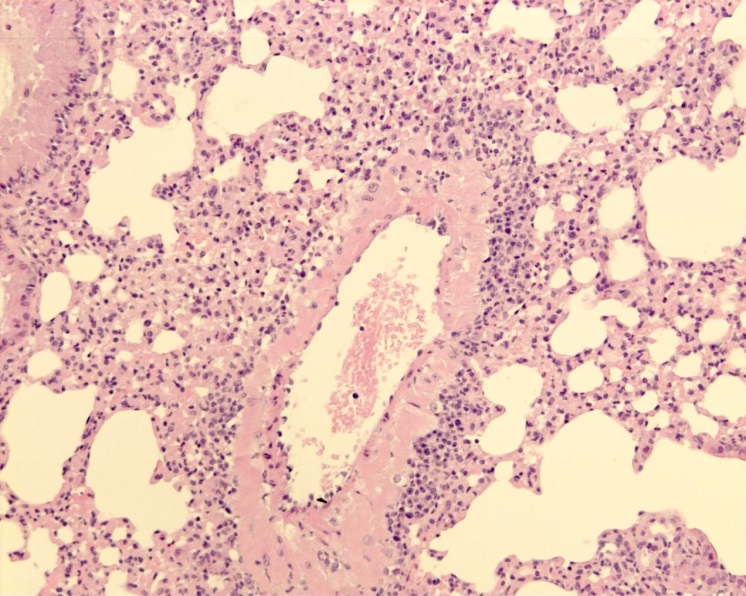


Control mIT


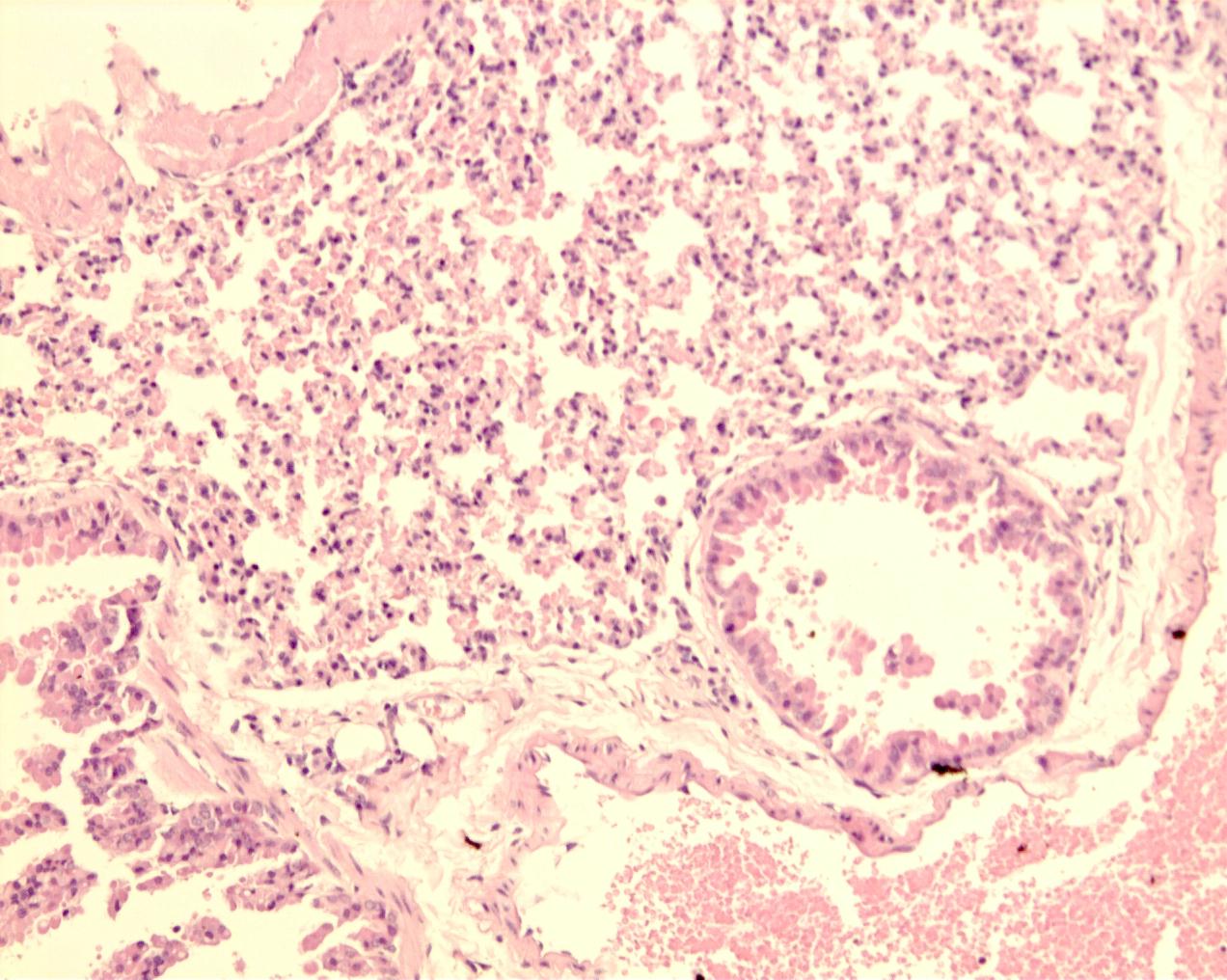

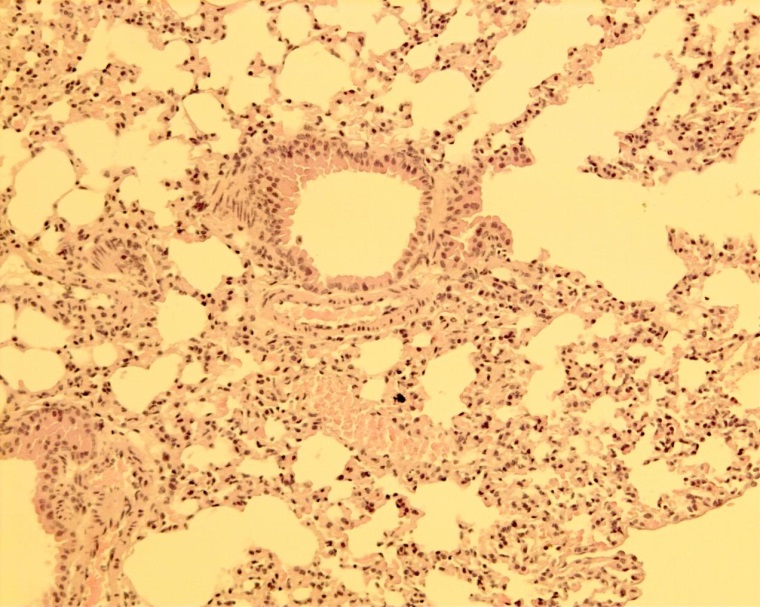


cIN alone cIN+mIT


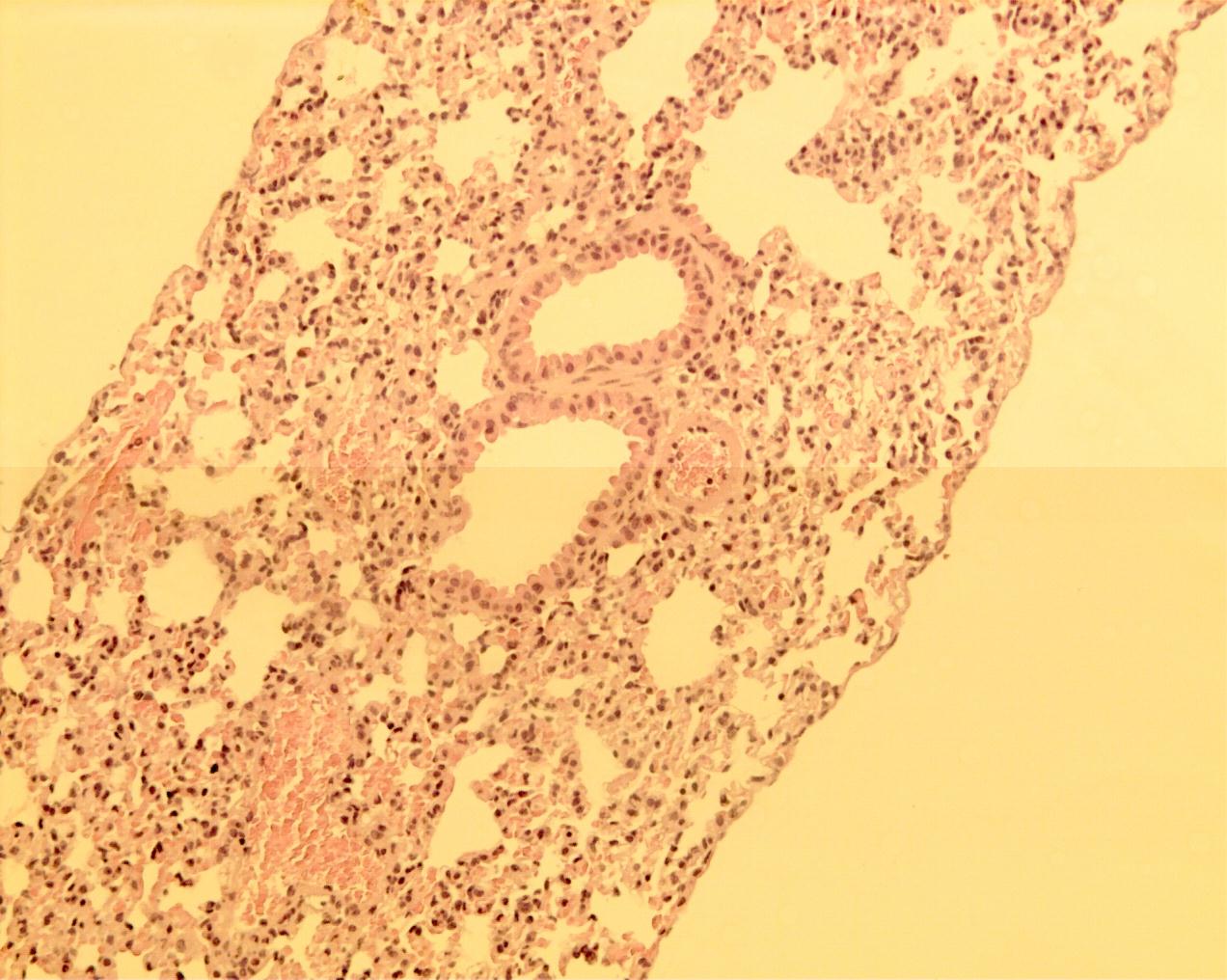

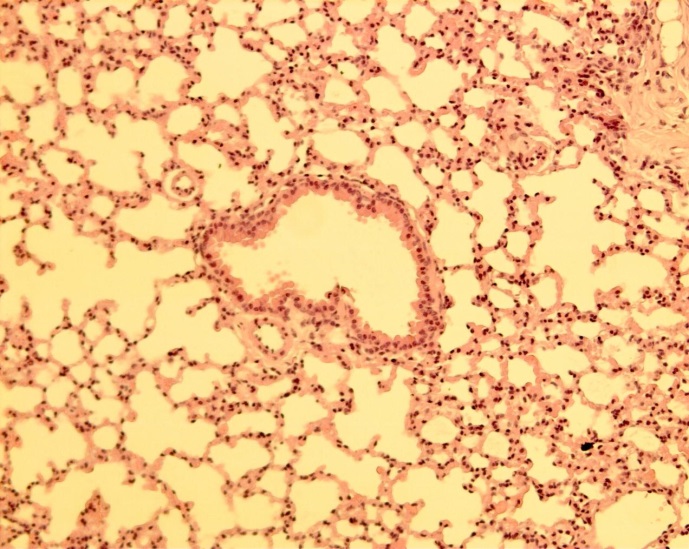


mIT+cIN cIN+mIT+cIN

PAS stain (light microscope 400x)


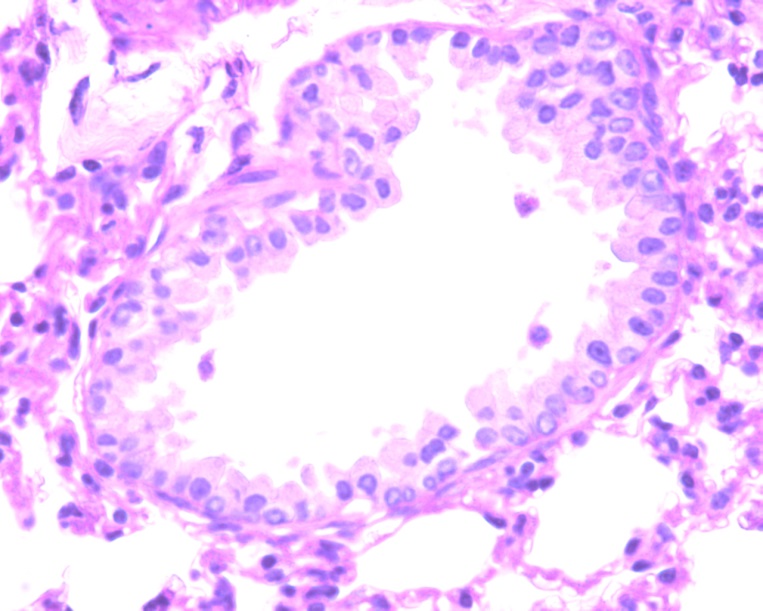

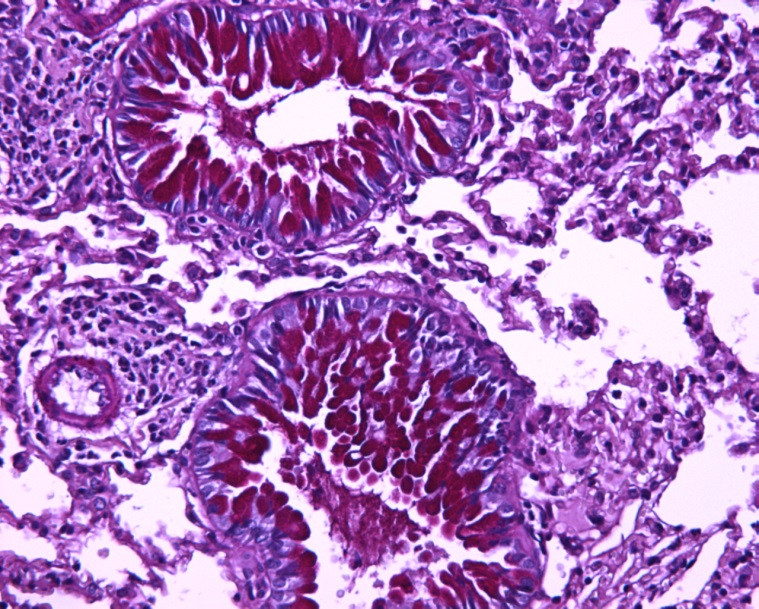


Control mIT


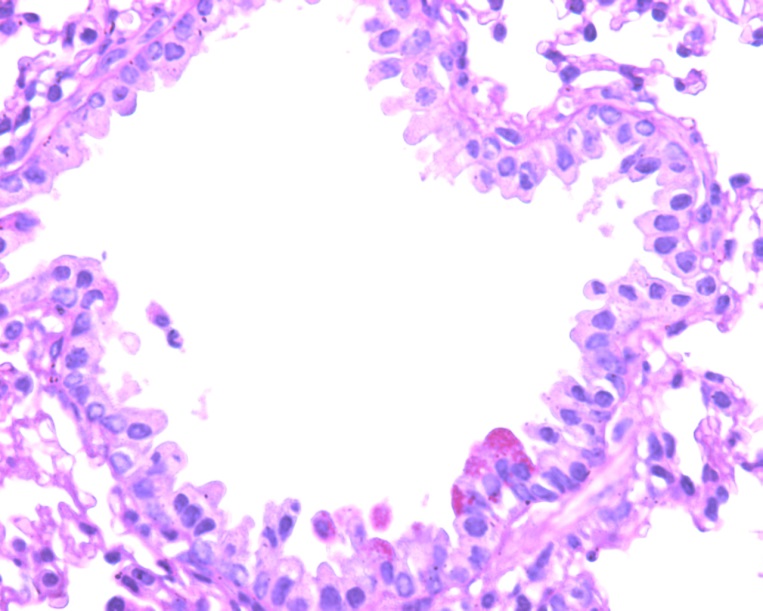

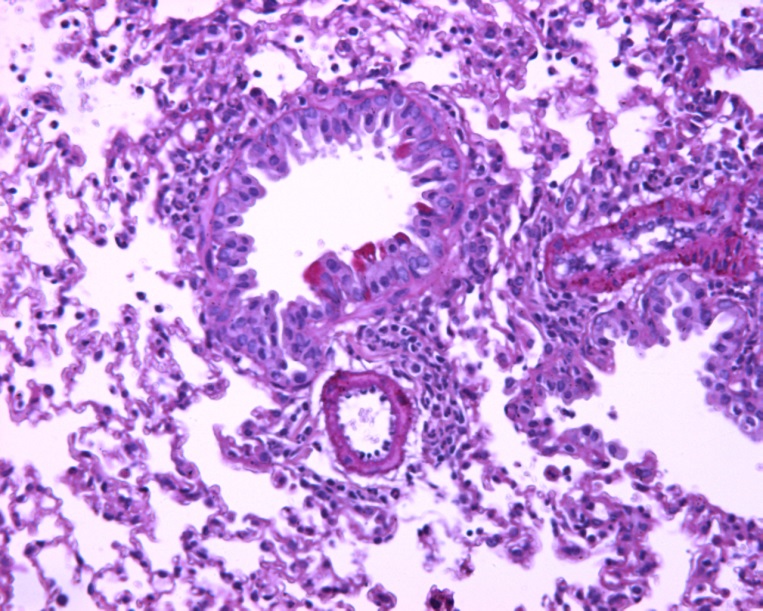


cIN cIN+mIT


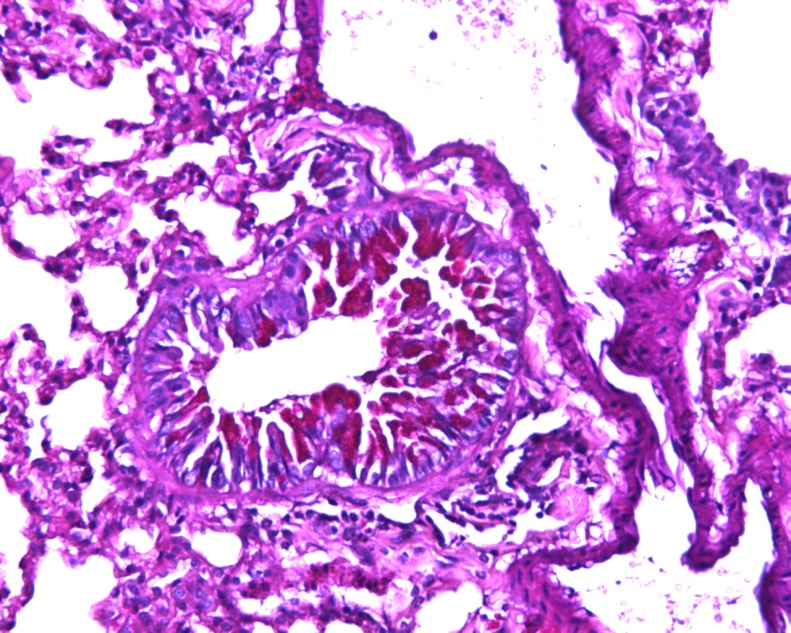

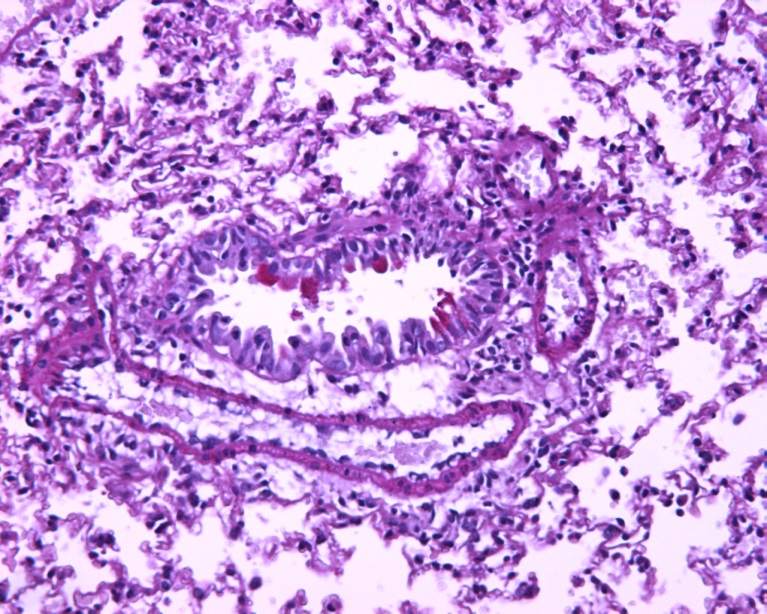


mIT+cIN cIN+mIT+cIN


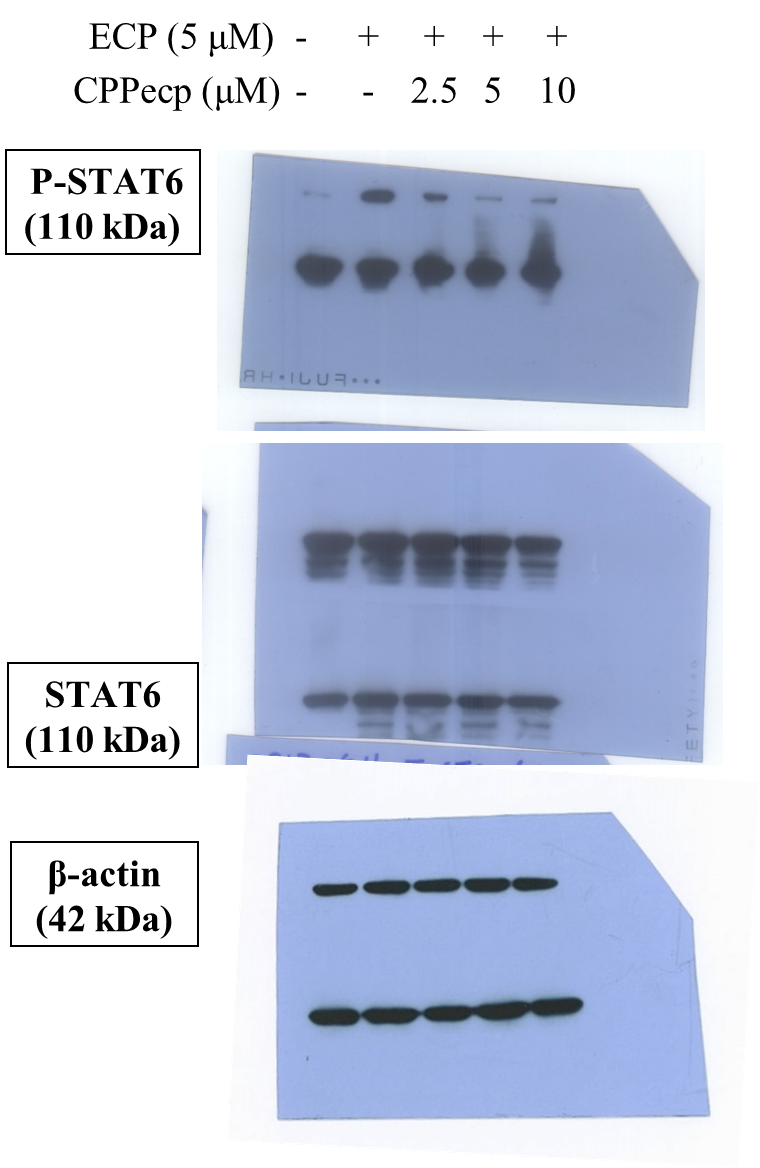
**Supplementary information 3: Original Western blotting**

**Figure S3-1 Original Western blotting film**

Original images of cropped blots of P-STAT6, STAT6 and β-actin are indicated in respective black arrows. All gels were run in the same experimental conditions (please see Supplementary information 1-Western blotting analysis of STAT6 and STAT6 phosphorylation for details).

**Fig. S3-2 Original Western blotting film**

Original images of cropped blots of P-STAT6, STAT6 and β-actin are indicated in respective black arrows. All gels
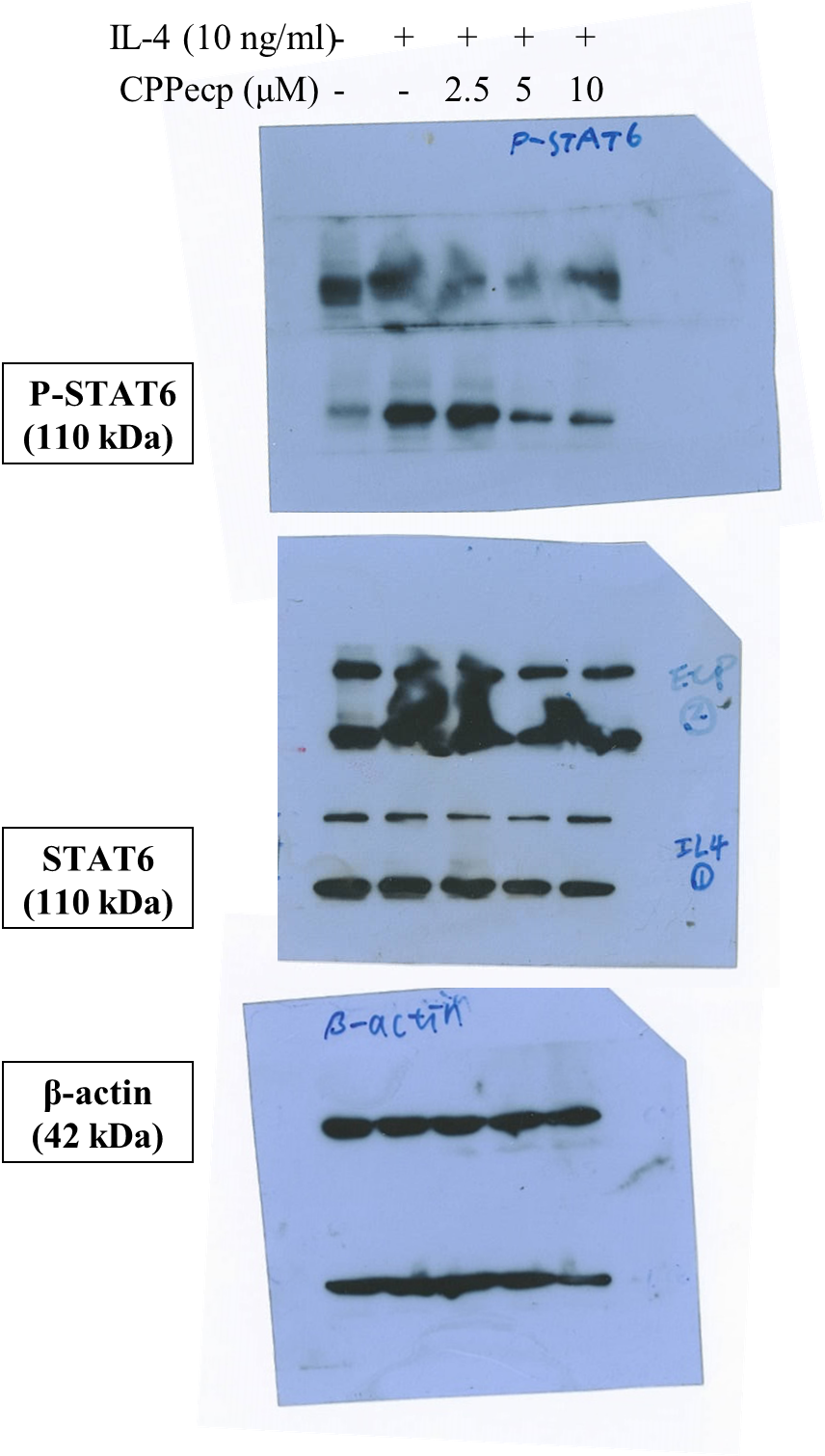
were run in the same experimental conditions (please see Supplementary information 1-Western blotting analysis of STAT6 and STAT6 phosphorylation for details).
